# Epidermoid cyst of the suprasternal region: a rare case report^☆^

**DOI:** 10.1016/j.bjorl.2016.04.010

**Published:** 2016-05-21

**Authors:** Manal Al Bin Manie, Khalid Hussain Al-Qahtani, Ahmed Al Ammar, Tahera Islam, Faiza N. Al Otaibi

**Affiliations:** a King Saud University, College of Medicine, Department of Otolaryngology-Head & Neck Surgery, Riyadh, Saudi Arabia; b King Saud University, College of Medicine and Research Center, Riyadh, Saudi Arabia

## Introduction

Epidermoid and dermoid cysts constitute the common benign masses that involve the skin. They are classified as epidermoid cyst when the lining contains only squamous epithelium, “true” dermoid cysts when skin adnexa such as hair follicles, sebaceous glands, and sweat glands are present and teratoid cysts when tissues from all three germ layers, such as cartilage, bone, muscle, and respiratory or gastrointestinal epithelium are present.^1^

These lesions may arise anywhere in the body. Dermoid and epidremoid cyst of the head and neck account for 7% of all dermoid epidermoid cysts.^2^ Suprasternal region is an uncommon site of presentation for epidermoid cyst with the first case documented in Turkey^2^ in 2008 and recently six in india.^1^ We report a case of an epidermoid cyst in the suprasternal region, presented as a large mass associated with odynophagia and weight loss.

## Case report

A 13-year-old boy, medically free, presented with swelling in the suprasternal region for more than 4 years. The swelling increased in size in the last one month prior to his presentation. Past medical history revealed an attack of upper respiratory tract infection aggravating the condition. The child had a history of painful swallowing for the one month, as well as weight loss of about 2–3 kg within the same period. There was no history of hoarseness of voice, shortness of breath or nasal obstruction. No history of trauma or previous surgical procedures. Family history was negative for similar condition.

Neck examination revealed 2.5 cm × 2.5 cm mass in the suprasternal region. The mass was firm, not tender, with no overlying skin changes discoloration or ulceration. The lower margin of the mass was difficult to palpate. No movement of the mass could be elicited with swallowing or protrusion of the tongue. No other neck swelling or lymphadenopathy noted. Systemic examination was unremarkable.

Routine laboratory investigations including complete blood count, liver function and renal function, all were normal.

**Figure 1:**
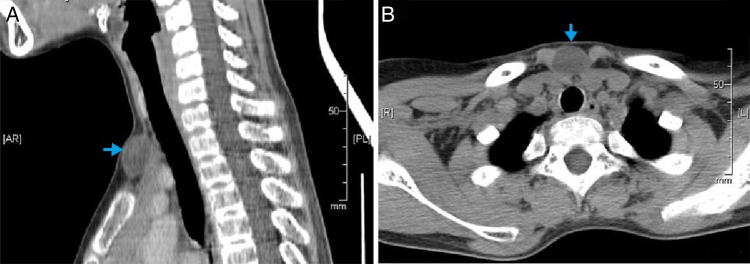
(A) CT neck sagittal showing the cyst (blue arrow) in the suprasternal area. (B) Axial contrasted CT scan of neck showing the cyst and its relation to the surrounding structure.

CT scan of the neck showed a cystic mass in the suprasternal region, not attached to deep structure, located in the midline, at the anterior side of the neck below the thyroid gland, and measuring 23 mm × 14 mm × 20 mm. It was very well defined and has fluid contents showing wall enhancement on contrast scan (Fig. 1A and B).

**Figure 2:**
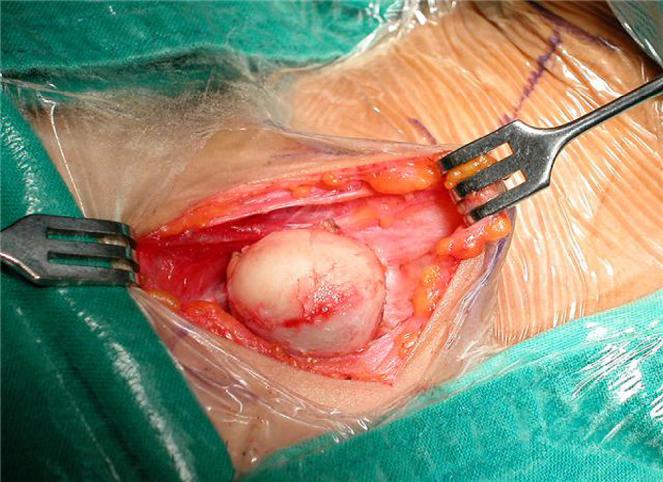
Intra operative view of the cyst and surgical dissection around it.

**Figure 3:**
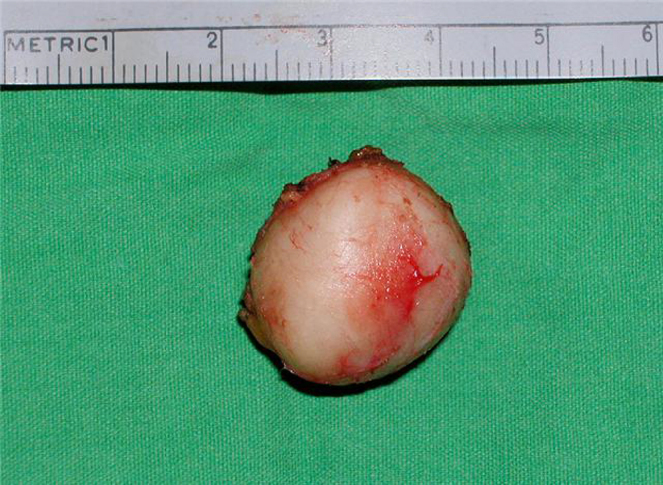
Intact epidermoid cyst after excision.

The cyst was removed by surgical excision under general anesthesia. A transverse incision was placed 2 cm above the sternum, dissecting around the capsule of mass (Fig. 2). Access to the lower margin was achieved by hyperextension of the neck and upward retraction of the mass. The mass was resected enbloc with intact capsule measuring about 2.5 cm × 2.5 cm and weighing 4.4 g (Fig. 3).

**Figure 4:**
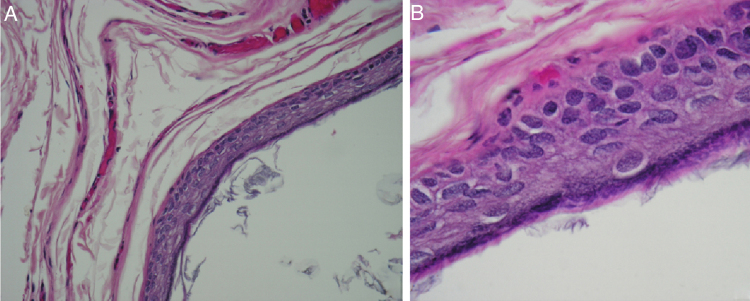
(A) Microscopic view of the epidermoid cyst. Cyst wall lined by keratinizing stratified squamous epithelium and contains keratin flakes (Hematoxylin and Eosin stain, magnification 100×); (B) microscopic view of the epidermoid cyst. Cyst wall lined by keratinizing stratified squamous epithelium (Hematoxylin and Eosin stain, magnification 400×).

Histopathologic examination demonstrated a cyst in the dermis lined by stratified squamous epithelium with keratinization. No adenexal structures (hair follicles or eccrine glands) were present in the cyst wall. The epithelium contains granular cell layer. The keratin consists of lamellar eosinophilic flakes. No cells were identified. This appearance was consistent with epidermoid cyst (Fig. 4A and B).

The child was followed-up for 1 year with no recurrence.

## Discussion

Neck mass in children is a common lesion. However, suprasternal epidermoid cyst is very rare. Epidermoid and dermoid cysts can be congenital or acquired even if the presentation or histology is similar.^3^ Congenital cysts are dysembryonic lesions that arise from ectoderm elements entrapped during fusion of the first and second branchial arches.^3^ Acquired cysts derived from traumatic or iatrogenic inclusion of epithelial cells or from the occlusion of a sebaceous gland duct. Dermoid cyst of the head and neck are thought to be congenital inclusion cyst.^3^

In exception of very few cases (Bowen's disease, Paget's disease, and squamous cell carcinoma), they are generally benign lesions.^2^

Subcutaneous epidermoid cysts are usually very small and commonly diagnosed clinically without imaging. Rarely, they grow sufficiently large to necessitate additional workup. Large or medium epidermoid cysts might be located at unusual sites close to specific organs mimicking tumors originating from that tissue as in our case.^4^

The natural history of epidermoid cysts is slow and progressive growth and they remain asymptomatic unless they enlarge in size. The rapid growth of the cyst may indicate either an infectious processes or malignancy.^5^ Squamous cell carcinoma arising from epidermoid cyst, although rare, has been reported in 10 cases.^6^

The weight loss observed in our case was not related to a neoplastic process, as proved by histopathology. We believe the weight loss in our patient resulted from poor feeding due to the painful swallow caused by this large cyst.

The differential diagnosis of anterior neck mass in children includes thyroglossal duct cyst, thyroid mass, cystic hygroma, cervical bronchogenic cyst and ectopic thymic mass.^7^

Surgical excision of epidermoid cyst is the treatment of choice.^8^ Usually, as in our case it is done in the operating room and under general anesthesia. But, office based surgical management with local anesthesia was found to be successful in small epidermoid cyst in different anatomic positions.^9^ In addition, the use of carbon dioxide laser has been reported in the treatment of multiple small epidermoid cysts.^10^

## Conclusion

Epidermoid cyst involving the suprasternal region is an extremely rare condition. So they might be misdiagnosed as malgninancy, especially when presenting with ominous sign like recent weight loss. Epidermoid cyst should be included in the differential diagnosis of cystic lesion of the neck especially in the young age group.

## Conflicts of interest

The authors declare no conflicts of interest.
